# Phenological data of a Neotropical savanna community

**DOI:** 10.1016/j.dib.2022.108560

**Published:** 2022-08-30

**Authors:** Adriano Valentin-Silva, Vinícius Nunes Alves, Priscila Tunes, Geissianny Bessão de Assis, Elza Guimarães

**Affiliations:** a Graduate Program of Biological Sciences (Botany), Institute of Biosciences, UNESP – São Paulo State University, Rua Prof. Dr. Antonio Celso Wagner Zanin, s/n, Botucatu, São Paulo 18618-689, Brazil; b Undergraduation in Biological Sciences, UNESP – São Paulo State University, Rua Prof. Dr. Antonio Celso Wagner Zanin, s/n, Botucatu, São Paulo 18618-689, Brazil; c Universidade Federal de Mato Grosso do Sul, Campus de Três Lagoas, Av. Ranulpho Marques Leal, 3484, Mato Grosso do Sul, Três Lagoas 79620-080, Brazil; d Laboratory of Ecology and Evolution of Plant-animal Interactions, Institute of Biosciences, UNESP – São Paulo State University, Rua Prof. Dr. Antonio Celso Wagner Zanin, s/n, Botucatu, São Paulo 18618-689, Brazil

**Keywords:** Cerrado, Flowering, Fruiting, Savanna, Sprouting

## Abstract

Savanna plant species commonly have different adaptive mechanisms in response to fire. In this biome, phenology is a functional trait characterizing the responses of plant communities to fire. The database presented here provides phenological data on 95 angiosperm species, in plots with natural vegetation and/or in burned plots. We used 10 plots (5 × 5 m) installed in “campo cerrado” physiognomy, in the Santa Bárbara Ecological Station, located in the municipality of Águas de Santa Bárbara, São Paulo state, southeastern Brazil. Half of these plots was burned in Aug/2013 and the other half was kept intact as a control. For one year (Sep/2013 to Aug/2014), we collected monthly data on the presence of sprouting (new branches or new unexpanded leaves), flowers (flower buds and flowers at anthesis) and fruits (immature and mature) in all angiosperm individuals present in the plots. This phenological data can support other studies on these sampled species, involving different aspects of their ecology, and on the conservation of this type of vegetation and management plans in relation to the prescription of fire.

**Specifications Table**

**Table:** 

Subject	Ecology
Specific subject area	Effects of fire on plant phenology
Type of data	Tables Graphs
How the data were acquired	We obtained the data in an area of “campo cerrado” vegetation in Brazil. We observed individuals in the field to assess the presence of phenophases, recording the data in spreadsheets.
Data format	Raw Analyzed
Description of data collection	We used 10 plots (5 × 5 m) installed in “campo cerrado” vegetation. Half of these plots was burned in Aug/2013 and the other half was kept intact as a control. For one year (Sep/2013 to Aug/2014), we monthly evaluated the presence of sprouting (new branches or new unexpanded leaves), flowers (flower buds and flowers at anthesis), and fruits (immature and mature) in all angiosperm individuals present in the plots.
Data source location	Institution: Santa Bárbara Ecological Station City: Águas de Santa Bárbara, São Paulo state Country: Brazil Latitude and longitude for collected data: 22° 41′–22° 46′ S, 49° 10′–49° 16′ W
Data accessibility	Repository name: Mendeley Data Data identification number: 10.17632/wt84rf8g2t.2 Direct URL to data: https://data.mendeley.com/datasets/wt84rf8g2t/2
Related research article	A. Valentin-Silva, V.N. Alves, P. Tunes, E. Guimarães, Fire does not change sprouting nor flowering, but affects fruiting phenology in a Neotropical savanna community, Flora 283 (2021) 151901. https://doi.org/10.1016/j.flora.2021.151901

## Value of the Data

• This database provides phenological data on 95 angiosperm species, in plots with natural vegetation and/or in burned plots. Plant species in savannas commonly have different adaptive mechanisms in response to fire. In this biome, phenology is a functional trait characterizing the responses of plant communities to fire.
• Information on plant phenology can support other studies on these sampled species, involving different aspects of their ecology. They can also be used by conservationist decision-makers, and policy makers dedicated to the management of protected areas in the Cerrado Domain.
• Future research may be carried out on biotic interactions of a plant species (or a set of species) with herbivores, pollinators, and dispersers. It also allows the comparison of the phenological responses of these plants with other savanna communities, considering the effects of fire. Finally, it can support studies on the conservation of this type of vegetation and management plans in relation to the prescription of fire.

## 1 Data Description

Our database consists of phenological data on vegetative (sprouting) and reproductive (flowering and fruiting) phenophases of species that occur in savanna vegetation in Brazil, which is available as .xlsx file in the repository Mendeley Data [1]. The ‘Species’ worksheet contains the floristic list with representatives of 38 angiosperm families, totaling 95 species. It also shows the mean and amplitude of the number of individuals sampled for each species. Not all individuals sampled in the plots presented the evaluated phenophases. The phenological worksheets present the presence/absence of phenophases and the number of individuals in plots where the vegetation was burned (Fire) and in intact plots (Control), over 12 months (Sep-2013 to Aug-2014). The data are organized by separating plant species according to the stratum of vegetation in which they occur, that is, in the herbaceous-subshrub layer or in the shrub-tree layer.

On average 2,308 individuals were sampled per month (Table 1). The herbaceous-subshrub layer was more representative, both in number of species (55.8%) and individuals (62.2%), than the shrub-tree layer (Table 1). Of all the species, 64.2% occurred in both treatments, which corresponds to 93.6% of individuals (Table 1). Of the species that occurred exclusively in the intact plots (“control” treatment), 75% (81% of the individuals) were from the shrub-tree layer (Table 1). In the burned plots (“fire” treatment), 55.6% of the species (64.3% of the individuals) that occurred solely in this treatment were from the herbaceous-subshrub layer (Table 1).

**Table 1:** Number of species and average number (amplitude) of individuals sampled in “campo cerrado” vegetation at Santa Bárbara Ecological Station, southeastern Brazil (*N* = 5 plots/treatment). Community values represent the sum of vegetation strata (herbaceous-subshrub and shrub-tree) values. C = control, F = fire. C/F = both treatments.

	Species				Individuals			
	C	F	C/F	Total	C	F	C/F	Total
Herbaceous-subshrub layer	4	10	39	53	12 (3–21)	54 (12–139)	1,370 (438–2,634)	1,436 (453–2,794)
Shrub-tree layer	12	8	22	42	51 (16–143)	30 (10–82)	791 (228–1,415)	872 (254–1,640)
Community	16	18	61	95	63 (19–164)	84 (22–221)	2,161 (666–4,049)	2,308 (707–4,434)

**Fig. 1:**
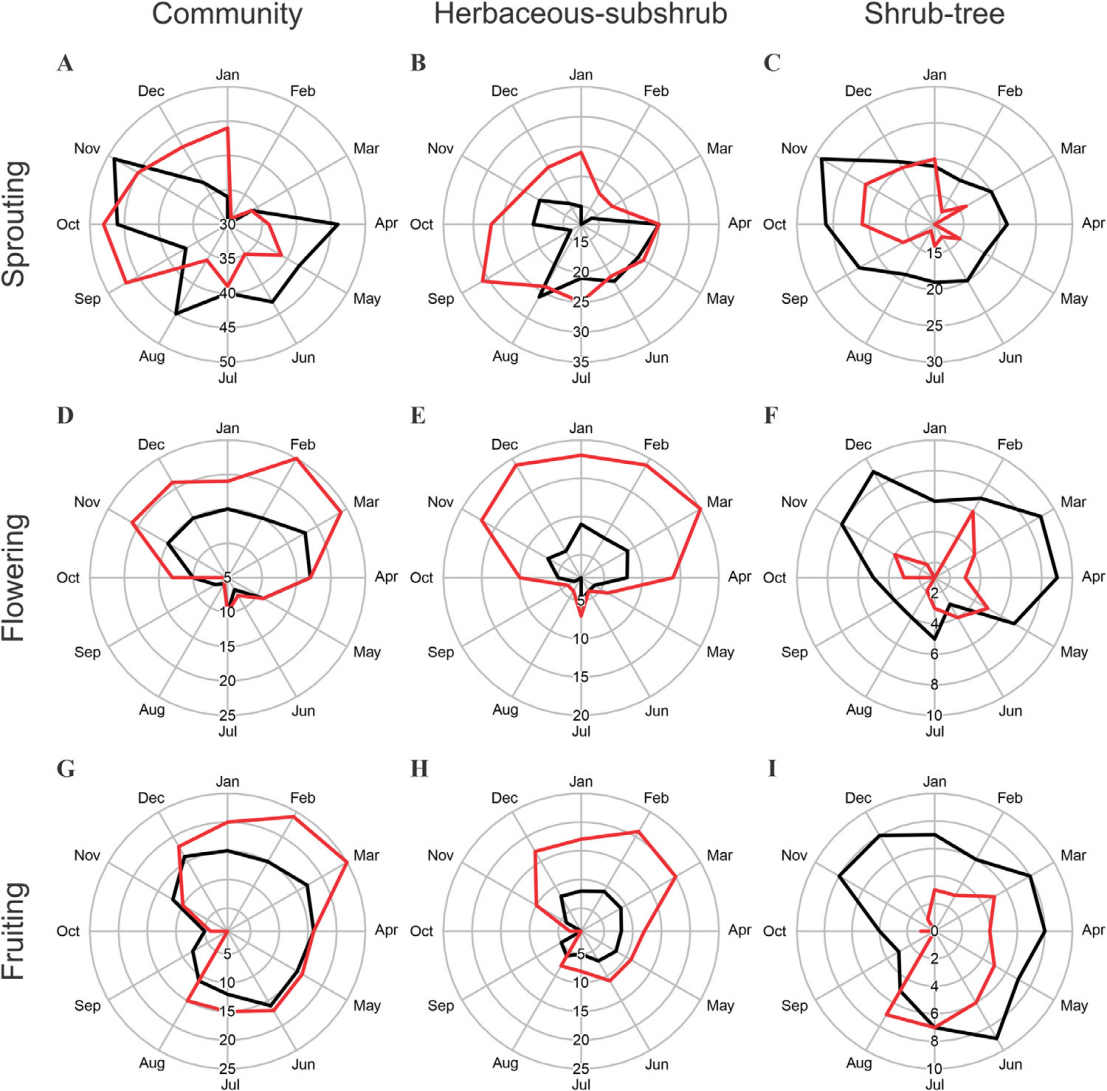
Plant phenology in ‘campo cerrado’ vegetation at Santa Bárbara Ecological Station, southeastern Brazil, considering the 95 species sampled in the area. Number of species expressing the phenophases sprouting (A–C), flowering (D–F), and fruiting (G–I), in the 'control' (black line) and 'fire' (red line) treatments.

We created circular graphs containing the number of species expressing each phenophase throughout the year (Fig. 1). Each graph compares the phenological responses of plant species submitted to “fire” and “control” treatments. The analyzes were performed at the community level (all species sampled together) and by vegetation stratum. Here we present the raw data used by [2] to examine the effects of fire on the period of occurrence, seasonality, and number of species sprouting, flowering, and fruiting in a savanna. This study used only the species that were common to both treatments with at least three individuals sampled (44 species), but here we provide the phenological data of the 95 species present in the plots.

## 2 Experimental Design, Materials and Methods

### 2.1 Study Site

We collected data in an area of “campo cerrado” vegetation, one of the savanna physiognomies from the Cerrado Domain, which occurs in the Santa Bárbara Ecological Station (EEcSB, 22° 41′–22° 46′ S, 49° 10′–49° 16′ W), located in the municipality of Águas de Santa Bárbara, São Paulo state, southeastern Brazil. The “campo cerrado” consists of a stratum of sparse small trees and shrubs, as well as of a continuous and moderately open stratum of grasses, herbs, and subshrubs [3]. According to Köppen's classification, the climate of the region is Cwa (rainy summer and dry winter; [4]), with average annual rainfall varying between 1,000 and 1,300 mm, and the monthly mean temperature varying from 16 °C to 25 °C [5].

This protected area covers 2,712 ha, with an altitude varying between 600 and 680 m, with a predominance of red latosol [5]. Its vegetation consists of savanna (“cerrado” *sensu stricto* vegetation) and seasonal semi-deciduous forest; 528 native plant species and 12 invasive species were recorded in the area, with the “cerrado” *sensu stricto* being the most species-rich phytophysiognomy (156) [5]. There have been records of invasion by exotic grass (*Urochloa decumbens* (Stapf) R.D. Webster) since 1970 in the EEcSB [6]. In addition to the richness of plant species, the station houses several animals, including 59 species of mammals, 206 birds, 32 amphibians, 29 reptiles, and 10 fish [5].

The densification of phytophysiognomies is one of the threats to the EEcSB biodiversity, which may be related to the suppression of fire [5]. Therefore, scientific research on fire management and its effects on vegetation has been carried out in the area. At least 10 accidental fire episodes were recorded in the EEcSB, which occurred at different locations [5]. Between 1985 and 2017, in the studied area, there was a single fire episode in 2001, in addition to the small experimental burns that were part of the study (pers. comm., Giselda Durigan).

### 2.2 Experimental Design

The plots used are part of the sampling of the project “Invasão do campo cerrado por braquiária (*Urochloa decumbens*): perdas de diversidade e técnicas de restauração”, developed by Geissianny Bessão de Assis under the supervision of Dr. Giselda Durigan. We used 10 plots (5 × 5 m) at least 5 m apart from each other, which had no records of invasion by *Urochloa decumbens* [6]. From the 2nd to the 5th of August, 2013, half of the plots were burned (“fire” treatment”), while the unburned plots were set as the “control” treatment. For this, a 2 m wide strip around each plot was lined with water before fire was applied. The experimental burning was conducted by the fire brigade of the conservation unit, using drip torches, fire swatters, and backpack sprayer pumps. Fire was set to each of the five plots, one at a time, during the morning, with low wind speed, at an average temperature of 26 °C. Fire was started from the windward edge of the firebreak. After the burning of part of the vegetation, fire was started on the other side of the firebreak, on the leeward side, facilitating its control. Fire was allowed to consume all the flammable biomass in a plot. If fire did not extinguish on its own, it was put out after approximately 1 h.

### 2.3 Data Collection

From September 2013 (30 days after the fires) to August 2014 (360 days after the fires), we monitored individuals within each plot monthly. We considered an individual (ramet) to be any branch emerging from the soil with no visible connection to neighboring branches, at least 5 cm apart from each other. Ramets can be different individuals despite sharing the same genome [7]. Due to the large number of individuals of some species and their reduced size, especially in the burned plots, we did not mark the sampled individuals. In addition, some individuals were included throughout the sampling period in the burned plots, as their aerial part became visible only a few months after fire. In this way, all individuals present in each plot and in each month were examined, which is related to the variation (amplitude) in the number of individuals sampled (Table 1). Scientific names of species were confirmed according to the website "Flora e Funga do Brasil" [8]. Based on field observations and species descriptions [8], we separated species into herbaceous-subshrub and shrub-tree layers (vegetation strata) according to their growth form.

Three phenophases were evaluated: sprouting, flowering, and fruiting. We considered that a given plant sprouted when it produced new branches or new leaves with non-expanded leaf blades; both basal and aerial sprouting were registered. We considered as flowering only the presence of flowers at anthesis, but we also recorded data on the presence of flower buds to help define the flowering period, as the anthesis period is short in some species and may not be recorded in monthly observations. For fruiting, we counted plants with immature and mature fruits in the same phenophase. Fruits in which the seeds had already been dispersed were used as an indication of the end of fruiting. We observed the presence and absence of vegetative (new branches and/or leaves) and reproductive (flowers and fruits) structures in all individuals and counted the number of angiosperm species that were expressing the evaluated phenophases. Field observations were recorded on paper worksheets, which were later converted to the online version (see [1]).

## Ethics Statements

Not applicable.

## CRediT authorship contribution statement

**Adriano Valentin-Silva:** Conceptualization, Data curation, Formal analysis, Methodology, Software, Supervision, Validation, Visualization, Writing – original draft, Writing – review & editing. **Vinícius Nunes Alves:** Data curation, Formal analysis, Investigation, Visualization, Writing – original draft. **Priscila Tunes:** Data curation, Investigation, Writing – review & editing. **Geissianny Bessão de Assis:** Conceptualization, Writing – review & editing. **Elza Guimarães:** Conceptualization, Funding acquisition, Methodology, Project administration, Resources, Supervision, Validation, Writing – review & editing.

## Declaration of Competing Interest

The authors declare that they have no known competing financial interests or personal relationships that could have appeared to influence the work reported in this paper.

## Data Availability

Phenological data of a Neotropical savanna community (Original data) (Mendeley Data)
